# Early neurophysiological indices of second language morphosyntax learning

**DOI:** 10.1016/j.neuropsychologia.2016.01.001

**Published:** 2016-02

**Authors:** Jeff Hanna, Yury Shtyrov, John Williams, Friedemann Pulvermüller

**Affiliations:** a Brain Language Laboratory, Department of Philosophy and Humanities, Freie Universität, Berlin, Germany; b Center of Functionally Integrative Neuroscience, Aarhus University, Denmark; c Department of Theoretical and Applied Linguistics, University of Cambridge, United Kingdom; d Medical Research Council - Cognition and Brain Sciences Unit, Cambridge, United Kingdom

**Keywords:** L2 acquisition, Morphosyntax, ERP/F, MEG, MMN

## Abstract

Humans show variable degrees of success in acquiring a second language (L2). In many cases, morphological and syntactic knowledge remain deficient, although some learners succeed in reaching nativelike levels, even if they begin acquiring their L2 relatively late. In this study, we use psycholinguistic, online language proficiency tests and a neurophysiological index of syntactic processing, the syntactic mismatch negativity (sMMN) to local agreement violations, to compare behavioural and neurophysiological markers of grammar processing between native speakers (NS) of English and non-native speakers (NNS). Variable grammar proficiency was measured by psycholinguistic tests. When NS heard ungrammatical word sequences lacking agreement between subject and verb (e.g. *we kicks), the MMN was enhanced compared with syntactically legal sentences (e.g. *he kicks*). More proficient NNS also showed this difference, but less proficient NNS did not. The main cortical sources of the MMN responses were localised in bilateral superior temporal areas, where, crucially, source strength of grammar-related neuronal activity correlated significantly with grammatical proficiency of individual L2 speakers as revealed by the psycholinguistic tests. As our results show similar, early MMN indices to morpho-syntactic agreement violations among both native speakers and non-native speakers with high grammar proficiency, they appear consistent with the use of similar brain mechanisms for at least certain aspects of L1 and L2 grammars.

## Highlights

• Used the syntactic mismatch negativity (sMMN) as brain index of grammar sensitivity.
• sMMN in proficient L2 learners resembles that of native speakers.
• sMMN is fast and requires no attention to elicit; reflects early, automatic grammar.
• Language skill assessed by psycholinguistic measures; reflect real time performance.

## 1 Introduction

The acquisition of a second language (L2) after childhood is famously difficult. Arguably, mastery of the grammar presents particular problems, while lexical learning remains relatively flexible in comparison (see Eubank and Gregg (1999) and Ullman (2004) for discussion). Understanding how and why this might be the case is one of the more enduring questions in the linguistic and neuro/cognitive sciences. Much neurophysiological research in this field has utilised the event-related potential/field (ERP/F) technique, which provides the temporal resolution necessary to follow linguistic processes as they unfold in real time. We describe here a study that uses the syntactic mismatch negativity (sMMN/m), an ERP/F which is fast, attention independent, and specific to linguistic information type (qualitatively distinct), to explore the neural substrates of grammar in native speakers of English (NS) and non-native speakers of English (NNS) of varying proficiency. Our results show that, among NNS, level of grammatical proficiency in processing subject–verb agreement-as assessed by experimental psycholinguistic methods-is reflected in this brain response.

Previous neurophysiological work on L2 syntax acquisition has mostly used one or more of three event-related potentials, each of which is thought to index aspects of syntactic processing: the early left anterior negativity (ELAN) appearing at around 150 ms after critical stimulus onset, the left anterior negativity (LAN) at around 300–500 ms, and the slow positive shift, or P600, at around 300–900 ms (Kutas et al., 2006). Much of the research has focussed on the question of whether “age of arrival” (AoA) or the acquired proficiency in the L2 is the primary determiner of neural representations of the L2, with the assumption that “nativelike” L2 learning is indexed by the ERP/Fs of learner groups converging with those of NS, as either AoA decreases, or proficiency increases, respectively. A few studies have favoured the AoA hypothesis (Weber-Fox and Neville, 1996, Pakulak and Neville, 2011), while a larger number found that NNS who began acquiring their second language later in life (typically after puberty), but who had nonetheless attained a high level of grammar proficiency, showed ERP responses resembling those found in native speakers (Bowden et al., 2013, Dowens et al., 2011, Frenck-Mestre et al., 2008, Hahne et al., 2006, Ojima et al., 2005, Rossi et al., 2006, Steinhauer et al., 2009). A handful of studies found that ERP responses were modulated by both AoA and proficiency (Dowens et al., 2009, Hahne, 2001, Hahne and Friederici, 2001). The results of this majority of studies converges with suggestions that grammar proficiency might not primarily depend on the age when language is learnt, but, instead, on other factors, including motivation and sociocultural embedding (Schumann et al., 2004), level of education (Dabrowska and Street, 2006), amount of learning (DeKeyser, 2007) and so on (Abrahamsson and Hyltenstam, 2009, Birdsong, 2006, DeKeyser, 2013 for discussion).

Recent studies have investigated initial stages of L2 language learning and employed multiple within-subject testing in the course of learning. A common finding of such work is that the P600 responses to grammatical errors develops with grammar learning (Osterhout et al., 2006), an effect which may appear as early as on the first day of training (Davidson and Indefrey, 2009). Longitudinal studies have observed L2 learners producing larger P600s with increasing L2 proficiency (White et al., 2012), with one study showing that participants at initial stages of learning reacted to syntactic errors with a posteriorly negative-going ERP pattern typical of semantically anomalous language (N400), which changed to the P600 response typically seen in native speakers when a higher level of proficiency had been achieved (McLaughlin et al., 2010;) Foucart and Frenck-Mestre (2012) found a differential pattern to grammatical violations, where both native speakers and learners of French produced P600s to gender agreement violations in noun–adjective pairs, whereas only native speakers produced P600s to gender mismatches in the less canonical adjective–noun pairs. Similar to McLaughlin's early stage learners, these L2 learners produced N400s to mismatching adjective–noun pairs instead. These results from learning studies lean toward a difference between late learnt and native grammar processes, which, however, may disappear with higher achieved proficiency.

On the surface, these findings appear consistent with the position that the N400 reflects semantic processing, the P600 reflects syntactic processes, and in cases of early or less successful second language learning, N400s appear in response to syntactic errors and reflect the use of the semantic system to compensate for the weakness of the syntactic system. We should remark however that this account is somewhat complicated by the fact that many studies have found N400-like negativities in response to syntactic anomalies in NS groups (Nieuwland et al., 2013, Osterhout, 1997, Osterhout et al., 2004; Severens et al., 2008), or that P600-like positivities occur in response to semantic anomalies in sentences which are both syntactically correct and simple (Bornkessel-Schlesewsky and Schlesewsky, 2008, Brouwer et al., 2012, Kos et al., 2012, Nieuwland and Van Berkum, 2008, Van Petten and Luka, 2012). More critical still, when ERP response profiles to syntactic errors are observed at the level of individual participants rather than just group averages, it seems that they vary along an N400-P600 continuum, similar to what was found in some of the longitudinal L2 acquisition studies.

What causes this variation is not yet clear, as recent attempts to find reliable correlations have produced unclear results. In one case, it was found that NNS participants who had lived in the L2 country longer and reported higher motivation to learn the L2 tended away from N400s and towards P600s in response to syntactic errors. However, in this study the motivation variable, which was at ceiling and thus lacked variance, was inappropriately dichotomised into high motivation and very high motivation. The authors found that L2 proficiency determined only the overall magnitude of the ERPs, rather than the balance between the N400 and P600 components (Tanner et al., 2013a). On the other hand, the same N400->P600-shift was found to correlate with proficiency in an NNS group (Tanner et al., 2013b), and also in an NS group in another study (Pakulak and Neville, 2010). In yet another case, the N400->P600 shift was modulated by dichotomised familial sinistrality in an NS group (Tanner and Van Hell, 2014). Given these quite variable results, which can be seen as at least partly incompatible with each other, it remains to be clarified what psychological processes are reflected by the N400/P600 complex.

Two observations indicate late brain responses such as the N400 or P600 might be a reflection of meta-linguistic processes and/or response preparation strategies to the behavioural tasks given in the experiments, rather than direct indexes of language understanding and parsing: First, attention to stimuli appears to be a necessary condition for full, robust N400 elicitation (e.g. Coulson and Brang (2010); for review Deacon and Shelley-Tremblay (2000) and Kutas and Federmeier (2011)). The P600 is also not reliably elicited when the stimuli are unattended, or even when they are attended but the accompanying behavioural task is not syntax-related (Batterink and Neville, 2013b, Gunter and Friederici, 1999, Hahne and Friederici, 2002). Second, the N400/P600 are late responses, generally peaking at 400–700 ms, and an extensive, decades-long body of behavioural research convincingly demonstrates that rapid-response latencies, often as early as 300 ms, are sensitive to the semantic and syntactic status of the inputs (Marslen-Wilson, 1973, Marslen-Wilson, 1985, Marslen-Wilson and Tyler, 1975, Moss, 1997, Sereno et al., 2003, Trueswell and Kim, 1998, Tyler et al., 2002, Zwitserlood, 1989). Subtracting the time necessary to plan and execute these behavioural responses, this means that semantic and syntactic integration of the input must have taken place extremely early, no more than 200 ms after the critical information was present in the input. Therefore, processes indexed by the N400 and P600 are two to three times slower than the earliest syntactic/semantic processes revealed by behavioural studies.

We seem then to be confronted with an ironic situation, where the great potential of neurophysiological methods for linguistic brain research – namely, that they make it possible to measure fast, automatic processes-is left unrealised by the use of ERP/Fs that are neither fast nor reliably automatic. This point becomes particularly salient in the measure of L2 acquisition, as rapid and automatic understanding of the L2 are intuitive and widely agreed upon hallmarks of successful acquisition. Unfortunately, matters are not much better when we consider the LAN. While it is arguably more robust against varying attention to stimuli (Batterink and Neville, 2013b), it can be generally unreliable to elicit (Tanner and Van Hell (2014), Tanner (2015); but see also Molinaro et al. (2011, 2015)).

Aside from the ERPs used, a further potential weakness in many L2 ERP studies is that measures of proficiency often do not include online psycholinguistic measures, instead mostly using self-ratings and/or offline, standardised language tests. The former measures are highly subjective, and the latter have two potential problems. First, the offline nature of tests allows the use of reflective, metalinguistic knowledge that would not be available in the rapid-fire context of actual language use. Second, language tests generally produce a unified measure of overall language competence, but L2 acquisition often proceeds unevenly across the various aspects of language (Eubank and Gregg, 1999, Long, 1990, Pulvermüller and Schumann, 1994, Schachter, 1996). One may for example have an extensive vocabulary or a good grasp of idioms, but little fluidity with grammar. An offline test, particularly a written one, allows the test taker to compensate for deficiencies in one area with strengths in another area. Therefore in addition to traditional measures, it is better to include online tests in measures of L2 proficiency, and preferably ones that correspond closely to what is being measured in the neurological experiment.

To address the issues of ERP timing and automaticity, we chose for our study a neurophysiological index which is a variant of the mismatch negativity, or MMN. The MMN is an ERP that was originally used as an index of auditory change detection (Näätänen et al., 2007), and is elicited by relatively rare “deviant” stimuli embedded in a train of frequently occurring “standard” stimuli. Compared with the standard stimuli, the deviants, differing in some acoustic feature such as loudness or frequency, elicit a fronto-central negativity in the event-related potential between 100 and 200 ms after stimulus onset. The MMN is elicited regardless of whether the participant attends to the stimuli or not, and has therefore been called “attention independent” by some authors, although attention levels may sometimes modulate its amplitude and topography. In fact, it is standard practice in MMN experiments to use visual material such as a silent film to passively distract participants from the auditory stimuli; in some cases even computer games or signal detection tasks are used to actively distract the participant while the MMN is elicited.

Crucially, when linguistic items are used as the stimuli, MMN amplitude, latency, and topography can be modulated not only by basic acoustic features, but also by abstract, linguistic features: phonological, lexical, semantic, morphological and syntactic (Pulvermüller and Shtyrov, 2006). With regard to the latter specifically, if a deviant stimulus is a linguistic unit (e.g., word stem or morpheme) embedded in an ungrammatical context, it produces a larger MMN than if the same deviant stimulus appears in a grammatical context. The extra MMN response amplitude to the ungrammatical deviant (compared with that to the grammatical one) is called the “syntactic MMN” (sMMN), or its magnetic counterpart (sMMNm). Because the crucial brain responses distinguishing between grammatical and ungrammatical contexts are typically elicited by the same deviant stimuli presented in the same type of paradigm but in different syntactic contexts (e.g., by the same word ‘runs’ in different contexts of the words “he” or “we”), this difference in brain responses cannot be explained as a consequence of acoustic stimulus features or short term memory processes alone, but allows for conclusions on genuine syntactic processes (for discussion, see Pulvermüller and Shtyrov (2006)).

The sMMN/m is an early response, normally starting ~100 after the recognition point of the critical word or morpheme, and its earliest part appears to emerge in the same way regardless of whether participants attend to the linguistic stimuli or not (Pulvermüller et al., 2008). The fact that the MMN can be elicited without subjects actively attending to critical stimuli is an important factor recommending its use in non-typical populations, for example in patients with attention deficits (Näätänen et al., 2011). As processing of a second language may be more attention demanding than L1 processing, standard attention demanding language tasks may be seen as requiring different attention levels in NS and NNS groups, thus opening the possibility for confounds in attentive reading or overt grammatical judgement tasks.

Although other brain indexes and tasks, including the ELAN and N400 along with their typical paradigms, undoubtedly index cognitive processes consistently present across both attend and non-attend conditions (Hahne and Friederici, 1999, Kiefer, 2002, Schiller et al., 2009), the MMN appears as a good candidate for the monitoring of brain correlates of language processes that are automatic in the sense that they do not require focused attention. Furthermore, the MMN can be related directly to neurobiological models of early language understanding and syntactic-semantic analysis, thus offering a neuromechanistic perspective on interpreting event-related brain activity (see, e.g., Pulvermüller (2010, 2013)). The electrical and magnetic MMNs indexing syntax and grammar have been recorded in Finnish (Shtyrov et al., 2003), English (Pulvermüller and Shtyrov, 2003, Pulvermüller et al., 2008), German (Hasting et al., 2007, Herrmann et al., 2009, Menning et al., 2005, Pulvermüller and Assadollahi, 2007), and French (Hanna et al., 2014). Here, we use the sMMNm to investigate brain correlates of morphosyntactic proficiency in NNS, specifically, brain responses to short English sentences with subject–verb agreement errors in both native English speakers and non-native speakers of English.

In order to address the above mentioned problem in previous studies, that language proficiency was only monitored by off-line tasks not tailored to crucial aspects of grammatical competence, we used well-established on-line psycholinguistic tests, such as cued shadowing and a timed, online grammaticality judgment task (GJT), which closely match the neurophysiological experiment in terms of the type of grammatical contrast tested. To find an optimal combination of online proficiency measures, we subjected these behavioural responses to principle component analysis, thereby producing an aggregate index of proficiency which was then linked to neurophysiological results through both categorical and correlation analysis. Critically, the grammatical contrast used in both psycholinguistic and neurophysiological tasks, namely subject–verb agreement, is not present in the L1 of the NNS group (Chinese). Therefore, any putative indices of grammatical processing seen in English NNS with L1 Chinese cannot be attributed to transfer of syntactic knowledge from their L1. We also obtained secondary proficiency measures using a modified version of the standardised English grammar test (TROG-E), and took note of what age participants were when they began learning English (age of onset, AO).

In summary we use psycholinguistic tests and a neurophysiological response, the fast, automatic sMMN/m, to measure syntactic proficiency and its putative brain correlates in NS and NNS groups. On the basis of past results cited above, we tested two hypotheses about the possible outcome: on the one hand (hypothesis I), the sMMNm of high proficient NNS may converge with the sMMNm of NS, who also have high proficiency, whereas low proficient NNS would produce smaller sMMNm. An alternative hypothesis (II) was that even high proficient NNS show distinct neural signatures from NS, and that despite their apparent proficiency their sMMNm response would cohere with those of the L2, so that brain response patterns would primarily reflect the NS vs NNS distinction.

## 2 Materials and methods

### 2.1 Participants

A total of 42 participants (predominately university students) took part in both behavioural and MEG experiments. Fourteen participants were native speakers of English (NS), and the remaining 28 participants were non-native speakers (NNS), from China. Table 1 displays mean age, reported age of English learning onset, and handedness according to the Edinburgh Inventory (Oldfield, 1971). Two NNS and one NS participants' source localisations produced extremely outlying voxel values, likely due to poor coregistration; therefore, data from these subjects were excluded from statistical tests on source analysis. All participants gave their informed consent and were paid for their participation. The study was approved by the Cambridge Psychology Research Ethics Committee.

### 2.2 Design

The presentation of stimuli followed the “optimum” or “multi-feature” MMN paradigm, in which frequently occurring standard stimuli and comparatively rarer deviant stimuli are presented alternatingly, with multiple deviants appearing in pseudorandom order, each preceded and followed by the same standard stimulus (Kujala et al., 2007, Näätänen et al., 2004). Our standard stimuli were two-word sentences, formed by a pronoun, followed by an uninflected verb (“he V” or “we V”, where “V” stands for a specific verb without any overtly realised affix, e.g. *we tick*). In one block of the experiment, all pronouns were “he”, and in the other block all pronouns were “we”. Deviant stimuli consisted of the given block-appropriate pronoun, combined with one of the three verb stems, but carrying an inflectional affix, either –s or –ed (see Table 2). Thus, a possible stimulus sequence of four could be “… we pick – *we kicks – we tick – we ticked …”. Note that, in the “we” block, the deviant stimuli carrying the –s affix are ungrammatical, whereas these same items are grammatical in the “he” block. These forms carrying the –s affix allowed for the main comparison of the experiment. Crucially, this comparison involved identical, overtly inflected verbs with the same suffix, thereby ruling out purely physical/acoustic confounds, while manipulating their grammaticality through the use of minimal context. The –ed deviants are grammatical in both blocks and therefore offer a control for the possible variance caused by the acoustically different pronouns. Verb stems were “tick”, “pick”, and “kick”. The order of stimuli was pseudo-randomised in accordance with recommendations from the previous literature (Näätänen et al., 2004), applying the following constraints: (1) a standard stimulus was followed by a deviant stimulus, and vice versa. (2) A group of 12 consecutive stimuli contained all 6 deviants once and each of the 3 standards twice. (3) No deviant stimulus occupying the last position in a series of 12 stimuli may stand in the first deviant position in the subsequent group of 12 stimuli.

There were 2400 presentations of stimuli in the experiment, 1200 per block. Each standard stimulus was presented 200 times, for a total of 1200 standard stimulus presentations. Each deviant stimulus was presented 100 times, for a total of 1200 deviant presentations, 600 for each suffix. Table 2 shows the possible combinations of morphemes into mini-sentences, listing all stimuli together with the probability of stimulus-type occurrence in a given block.

### 2.3 Stimulus recording and construction

The MMN is modulated by linguistic and other cognitive processes, but as it is primarily driven by acoustic change, meticulous control over acoustic stimulus features across conditions is essential to differentiate neurolinguistic effects from their neuroacoustic background. Thus, differing MMN dynamics across conditions can become interpretable as grammar indicators, rather than acoustic indicators. To assemble the 18 stimuli composed of pronouns (he and we), verbs (tick, pick, or kick) and inflections (–s, –ed, or no affix), the following phonemes and phoneme sequences were recorded within coherent linguistic contexts: [hi:], [wi:], [t], [p], [k], [I], word final [k], [ks], and [kt] (note that the “–ed” affix is realised as a devoiced [t] after unvoiced consonants, such as [k]). These were then isolated and spliced together to yield the 18 stimulus combinations. To ensure that no two constituents could combine more naturally than another pairs, all constituents were recorded in different contexts than the ones they would appear in during the experiment.

**Fig. 1:**
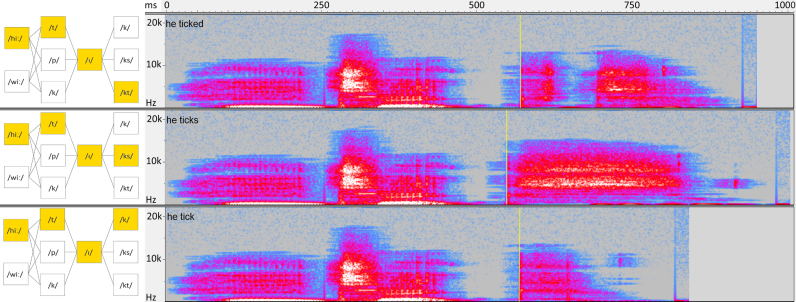
Examples of stimuli. Panels on the left show a schematic illustration of the phoneme combinations used in the experiments, with the constituent phonemes of the three displayed example items highlighted in yellow. Panels on the right show spectrograms of the respective audio information. The onset of the critical inflectional suffix (and zero point of epoch) is marked with a vertical, yellow line for each inflectional condition. (For interpretation of the references to color in this figure legend, the reader is referred to the web version of this article.)

A male, native British English speaker was recorded speaking sentences containing the required building blocks with a TASCAM HD-P2 super high-definition digital recorder (TASCAM, Wiesbaden-Erbenheim, Germany), in a soundproof booth, and the fundamental phonemic constituents were then isolated and matched for sound volume and length. These constituents were then combined to form the actual stimuli (Fig. 1 for examples). All sound editing was done with Audacity 1.2.6 (audacity.sourceforge.net). Before the experiment, two native speakers of English confirmed that all combinations were natural-sounding.

### 2.4 Procedure

#### 2.4.1 MEG data collection and preprocessing

Participants were fitted with four head position indicator (HPI) coils and, for recording eye-movements, horizontal and vertical electrooculogram electrodes. Their head shape was digitised using Polhemus Isotrack (Polhemus, Colchester, VT). They were then seated in the MEG chamber (Imedco CO., Switzerland) and fitted with non-magnetic ear-pieces, through which they heard the stimuli. During the stimulation, the participants neuromagnetic brain activity was recorded continuously (passband 0.03–300 Hz, sampling rate 1000 Hz) using a 306-channel MEG system (Elekta Neuromag, Helsinki, Finland) containing 102 sensor locations, each locations consisting of a magnetometer, and two planar gradiometers with orthogonal orientations. Continuous MEG, EOG and HPI signals were acquired. The experiment lasted approximately one hour, and the participants were provided with their choice of a film to watch without sound or subtitles. They were instructed to attend to the film, pay no attention to the auditory stimuli, and avoid movements or blinks. The two blocks of the experiment were counterbalanced across participants.

MEG data were pre-processed using temporal extension of the signal space separation (tSSS) technique (Taulu and Kajola, 2005), applied by the Maxfilter 2.0 utility (Elekta Neuromag), using HPI-based movement compensation, adjusted at every 200 ms, and spatiotemporal filtering in 4 s windows. Any bad channels were re-interpolated using the tSSS algorithm, and every participant's data were then transformed to a uniform coordinate space.

All pre-processing from this point forward was carried out using SPM12 (FIL, London, UK, http://www.fil.ion.ucl.ac.uk/spm/software/spm12). The data were first downsampled to 200 Hz, and then bandpass filtered at 0.1–30 Hz. Epochs were 900 ms long, consisting of a 150 ms baseline and an 800 ms post stimulus interval. Event-related brain responses were time-locked to the onset of the final consonants or consonant clusters [k], [ks], or [kt], as these were the first points in time when the grammatical status of the stimulus became apparent, as well as when the deviant stimuli diverged acoustically from the standards.

Epochs were averaged into event-related fields (ERF), using the robust averaging technique, where every given time point within a given condition is weighted across trials, based on its divergence from the median for that given time point. In this way, any noise which is not in phase with the stimuli is greatly reduced (Litvak et al., 2011). After this point, all lexemes within a pronoun-inflectional condition were averaged together, and so all distinctions between [tik], [pik], and [kik] were elided, while also maximising the number of trials used and correspondingly the signal-to-noise ratio (SNR) of the resulting ERFs. This produced a standard condition, and –s and –ed deviant conditions for both pronouns. Standards were then subtracted from their respective deviants to produce magnetic MMN (MMNm) ERFs for the four conditions of interest (he –s, he –ed, *we –s, and we –ed). The resultant four MMNm were analysed in both source and signal space.

Analysis of signal space focussed on planar gradiometer recordings. Each recording site contained two orthogonally oriented gradiometers, which were combined into a single measure by taking the square root of the mean of the squared signals from each gradiometer (root mean squares, or RMS), and applying baseline subtraction once again, to adjust for squared baseline noise. As is standard in MMN(m) research, comparison between conditions was constrained to time windows and sensors where the overall MMNm amplitude (i.e. average across all conditions) was strongest. Inspection of topographies and waveforms (see Fig. 3) showed clear bihemispheric centres of activation in left and right hemispheres, which we further divided into anterior and posterior sections, owing to morphological differences in their topography. Anatomically, these sensor regions were above left and right fronto-temporal cortex; they were also consistent with the topographies found in past sMMNm studies (see, e.g., Shtyrov et al. (2003), Herrmann et al. (2009), Garagnani et al. (2009) and MacGregor et al. (2012)). Each of the four topographical regions was composed of four gradiometer pairs, which were averaged together to form each region's RMS amplitude. Their precise locations are indicated visually by the parallelograms in Fig. 3b.^1^ The main time window for analysis was defined in a data driven manner (see Section 3).

For source space analysis, both magneto- and gradiometers were coregistered to a single-shell MEG forward model, based on SPM's canonical MRI, and ERFs were then inverted into source space using the Multiple Spare Priors (MSP) approach (Friston et al., 2008), with group-level prior constraints (Litvak and Friston, 2008). Inversion was constrained to the 80–335 ms time window, and MNI images were then produced, summarising source activity for each of the four conditions in this time window. The average values of 2 mm radius spheres of voxels, one placed at each hemisphere's maximally active voxel across conditions and participants (see Fig. 5a), were the dependent variables in statistical analysis in source space.

#### 2.4.2 Behavioural experiments

All participants who underwent the MEG experiment also took part in the behavioural experiments.

##### 2.4.2.1 Shadowing

Cued shadowing here involves asking participants to repeat (or “shadow”) words that are situated in sentential contexts which render them either grammatical or ungrammatical, and timing the repetition speeds with millisecond precision. For example, upon hearing stimuli such as “the chef bakes” or *“the chefs bakes”, the participant must repeat “bakes” as quickly as possible. Past research has demonstrated that participants are slower by several tens of milliseconds when repeating ungrammatical stimuli in comparison to grammatical stimuli (Akhutina et al., 2001, Akhutina et al., 1999, Bates et al., 1996, Guillelmon and Grosjean, 2001, Lu et al., 2001, Marslen-Wilson, 1985). The shadowing task does not require participants to be reflective or even aware of the grammatical status of the stimuli, and this, combined with small latency differences, suggests that speech shadowing measures a rapid, automatic syntactic competency. One study suggests that second language acquirers can be similarly delayed as native speakers when shadowing ungrammatical stimuli (Guillelmon and Grosjean, 2001). We therefore posit that a response latency gap between grammatical and ungrammatical stimuli is indicative of implicit syntactic proficiency in the language.

Participants were seated in a sound-isolating testing cubicle in front of a computer with a voice-key microphone positioned in front of them. They were told that they would be hearing sentences and were instructed to repeat the last word of the sentence as quickly and as accurately as possible. Participants were informed that the terminal word would be marked by the appearance of a fixation point on the screen as the word is played. They were also warned beforehand that some sentences would be ungrammatical, but that this should not affect their repetition of the sentence-final word. After the instructions, participants were given a practice round of eight trials after which further instructions were given if necessary. Stimuli were presented binaurally through headphones.

At the onset of the sentence-final word (the verb) in an individual trial, a fixation cross appeared on the screen, the voice key was activated, and the latency timing began. After the participant started to speak, thus triggering the voice key, the fixation cross immediately disappeared from the screen, and the next trial followed after a one second pause. The experiment took around 20 min to complete, and was administered with E-prime 1.2 (Psychology Software Tools, Sharpsburg, PA).

There were 84 trials, presented in a random sequence. Each trial consisted of a pronoun or a definite article and a noun, followed by a verb. One third of the trials had a verb with –s inflection, and another third used a verb with –0 inflection. Both –s and –0 inflection conditions were each further divided into trials with singular or plural subjects. Singular subjects are syntactically consistent with the –s inflection, but not the -0 inflection. With plural subjects, grammaticality-suffix relations are reversed. The final third of the stimuli used past tense verbs, which are syntactically compatible with both singular and plural subject, and so were used as fillers. Verb lexemes rotated around the five possible conditions, such that all participants heard the same lexemes, but the particular inflectional condition in which a given verb lexeme was realised varied across participants. This therefore prevented extraneous features of the lexeme (e.g. frequency, semantic associations) from biasing the condition averages. Participants completed the shadowing experiment, and then the GJT, and received a different verb lexeme rotation for the two experiments.

An experimenter was present in the room to note aberrant trials. Reasons for aberrant trials include false triggerings of the voice key, which could result either from inhalation or other non-linguistic noise, or from the triggering of the voice key by a non-initial phoneme. Occasionally participants reproduced a verb with an incorrect inflectional ending, and this was also marked as an aberrant trial. Aberrant trials were excluded from the analysis. Finally, latencies more than 2.5 standard deviations from the mean response time of a given participant were excluded.

##### 2.4.2.2 Grammaticality judgment task (GJT)

The timed GJT assesses participants' speed and accuracy in determining the grammatical correctness of local subject-verb pairs (the stimuli here are the same as in the shadowing task). There is a then a metalinguistic component to the task, in that participants are required to make explicit judgments about sentences and answer with a button press whether they are correct or not, though the fact that the task is performed under strong time pressure encourages participants to rely more on intuitions rather than deliberation. Both latency and accuracy measures are potentially useful, with more proficient participants likely to be more accurate and produce shorter latencies.

The GJT experiment was structured in a very similar fashion to the shadowing experiment. The same stimuli from the shadowing experiment were used, but with different inflectional status. Participants used a button press to indicate whether the sentence was grammatical. Latencies and accuracies were recorded; participants were instructed to respond correctly and rapidly, but speed was emphasised and subjects were explicitly encouraged to respond spontaneously and discouraged from thinking about the answer. Trials where the participant gave an incorrect answer were excluded from the latency analysis. Latencies more than 2.5 standard deviations from the overall participant mean were also excluded. Accuracies were converted into d-prime measurements.

##### 2.4.2.3 Test for the Reception of Grammar (TROG)

The TROG (Bishop, 2005) is a standardised test which measures grammatical competence by minimising the degree to which test takers can rely on semantic/pragmatic cues. Test-takers hear an English sentence, and see four pictures, only one of which corresponds to the correct meaning of the sentence. Picture choices differ from each other minimally such that only a correct parsing of the sentence structure and understanding of its grammar allows the test-taker to arrive reliably at the right answer. For example, in one item designed to test competence with relative clauses, the participant hears, “The man that is eating is looking at the cat”. The available pictures include a man who is eating and turned away from a cat, a man who is looking at an eating cat, a man carrying a box away from an eating cat, and the correct choice of an eating man looking at a cat.

TROG is designed primarily for the diagnosis of speech, learning, and hearing impairments in children learning English as their native language. For this reason, some modifications to the scoring system were necessary, and we therefore speak of “modified TROG” results below. First, TROG scores are typically adjusted for children's age (i.e. developmental stage), but since our participants were all adults, we used absolute scores, unadjusted for age. Second, TROG scores are usually calculated by blocks of four questions, rather than individual questions. Each of these mini-blocks tests a specific grammatical competency. Answering one question incorrectly causes that question's entire block to be marked as a failure. However, as late second language learners frequently acquire incomplete grammatical competence of their L2 (Johnson and Newport, 1989, Schumann, 1976), there was relevant information immanent to the number of errors made within each mini-block. Therefore we chose to score by individual question, rather than block. Finally, TROG allows the test taker to request repetitions of the sentence. Whilst observing our participants taking the test, however, it became clear that some of the less proficient performers were using the repetition option as a crutch, sometimes asking for more than a dozen repetitions on a single item. To correct for this, repetition requests counted as wrong answers. This study used the TROG-E version of the test, which is a computer administered version of the paper-based TROG-2 test.

## 3 Results

### 3.1 Handedness, age, and age of onset

Though all participants scored in the right-handed range on the Oldfield inventory (scale of 100L–100R), the laterality index of NS was almost 20 points more right-handed than that of NNS (see Table 1). This difference was statistically significant (*t*(40)=2.78, *p*=0.008). There were no significant differences between high and low proficient NNS groups either with handedness, AO, or age. The average numerical age of the NS group was 3 years lower than of the NNS group. The general high proficient group (NS+HP) was three years younger than the low proficient group and seventeen points more right-handed. These differences were statistically significant at *t*(40)=−2.23, *p*=0.03 and *t*(40)=2.4, *p*=0.02, respectively.

### 3.2 Behavioural results

**Fig. 2:**
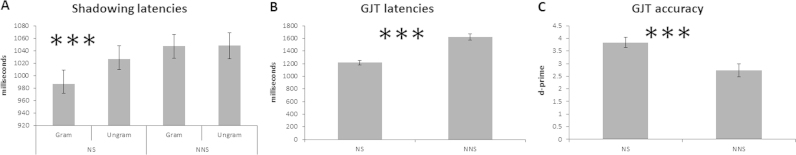
Means and standard error bars for behavioural tasks, A: shadowing experiment, B: GJT latencies, C: GJT accuracy (converted to d-prime).

The three online psycholinguistic measures of syntactic processing – GJT accuracy and latency, and cued shadowing performance-were each analysed separately. NS were reliably more accurate on the GJT than NNS (*t*(40)=3.73, *p*=0.001), as well as faster (*t*(39.63)=−6.25, *p*<0.001, d*f* adjusted for unequal variances). Results from the shadowing task were submitted to an ANOVA to assess group differences between natives and L2 speakers in processing grammatical and ungrammatical strings as well as the contribution of inflection (–s or –0) (design: NS/NNS (2 levels)×Grammaticality (2 levels)×inflection (2 levels)). For shadowing performance, there was a significant interaction of grammaticality and group (*F*(1,40)=11.2, *p*=0.002), due to a reliable difference between grammatical and ungrammatical conditions in NS (*F*(1,13)=19.18, *p*=0.001, *η*=.596), but not in NNS (*F*(1,27)=0.01). Interactions of inflection with grammaticality and group did not approach significance in any statistical test. Fig. 2 shows mean values and standard error for behavioural psycholinguistic measures.

Having established that a range of psycholinguistic measures allow for reliable group-level distinctions between NS and NNS, we checked pairwise correlations between these measures and found high correlations throughout Even though these grammar measures exhibit high correlations with each other, the most substantial *R*-values are around +/−.5. Therefore, each of them still captures substantial variance invisible to the others so that an integrative measure of grammar proficiency, taking into account evidence from linguistically-motivated grammatically judgment tasks and psycholinguistically-grounded shadowing might bring together their distinct contributions. Results were therefore combined using Principal Component Analysis (PCA) into a single measure that will be used as an index of proficiency. We submitted shadowing performance, GJT accuracy and GJT latencies for each of the 42 subjects who participated in both the MEG and behavioural experiments to a principle component analysis (PCA). The three components produced by the PCA had eigenvalues of 1.92, 0.72, and 0.37, which respectively accounted for 63.86, 23.89, and 12.25 percent of the variance in the data. Given that the latter two components failed the Kaiser criterion by having eigenvalues less than one (and were therefore less informative than a single variable), and given that the first variable already explains well over half the variance, we set the latter two aside and focussed on the first component, henceforth referred to as principal component one (PC1).

Unsurprisingly, PC1 correlated significantly with the difference in RTs between grammatical and ungrammatical items in the shadowing task (*r*(40)=.67, *p*<0.001), as well as with the GJT d’ (*r*(40)=.86, *p*<0.001) and latencies (*r*(40)=−.85, *p*>0.001). Better performance was reflected by higher PC1 values. Shadowing performance, GJT accuracy, and GJT latency contributed substantially to PC1, with a slight predominance of GJT measures (23.65%, 38.49%, 37.86%, respectively). The weaker contribution of shadowing performance may be related to the inherent variability of these data or to the fact that difference values were used (latency to ungrammatical items minus latency to grammatical items), which further decreases their signal to noise ratio.

These data strongly suggest that PC1 indexes syntactic proficiency. As an external validation of this interpretation, we examined the relationship between the errors participants made on the modified TROG with their values on PC1, finding a highly significant correlation (*r*(40)=−.8, *p*<0.001). The same correlation was significant when we restricted the data to NNS (*r*(26)=−71, *p*<0.001). These results show that those who placed highly on PC1 also tended to make less errors on modified TROG and confirms the validity of PC1 as a measure of grammar proficiency. We therefore used PC1 to subdivide the NNS group into low- and high-proficiency L2 users, on the basis of a median split of PC1, and as the basis for a correlational analysis on the source results.

### 3.3 MEG results

#### 3.3.1 Group division

For factorial analysis of ERF signal space results, we compared our group of NS with NNS with HP and LP. To this end, NNS participants were subdivided into high and low proficient groups (HP, LP) based on a median-split division, according to their behavioural linguistic performance as assessed by PC1, the psycholinguistic aggregate index of L2 grammatical proficiency (see Section 3.2).

#### 3.3.2 Signal space

**Fig. 3:**
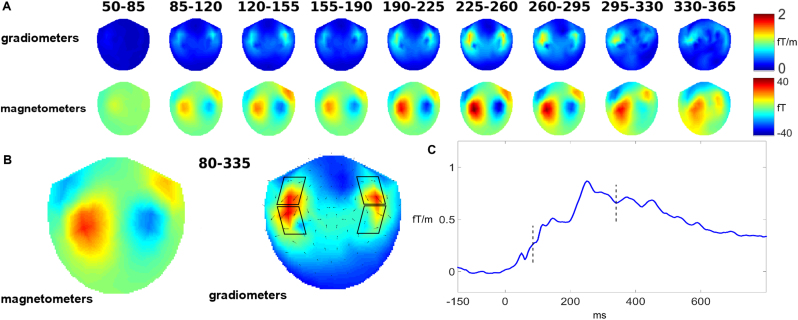
Time and topography of MMNm responses averaged across all conditions. A: MMNm topographies as shown by planar gradiometer (local RMS values calculated from gradiometer pairs are displayed in fT/m) and magnetometer recordings (in fT) in the first 365 ms after acoustic divergence. B: Average topography of the MMNm in the time window 80–335 ms, as recorded through magnetometers and gradiometers. Arrows on the latter reflect direction of field gradients. Parallelograms index gradiometers delivering large signals in the present task, which were therefore selected for further signal space analysis. Each parallelogram indicates a group of sensors whose average formed one region of interest in the sensor-space ANOVA. C: Time course of the MMNm response recorded through all large signal gradiometers (RMS values from ROIs indexed in b). Borders of time window used for signal space analysis, which capture both early MMN peaks, are indicated by vertical dashed lines.

Visual inspection of topographies revealed that, in agreement with the previous literature, the MMNm response picked up in the MEG gradiometers was largest above bilateral fronto-temporal regions (Fig. 3a and b). The RMS signals calculated from these high-amplitude waveforms show a biphasic MMNm response, with an early relative maximum at 145 ms, and a major peak 255 ms, followed by the first relative minimum at 335 ms. Thereafter, the MMN curves slowly returned towards the baseline, with small relative maxima around 400 ms. Because the MMN is known to be an early brain response peaking within the first 100–300 ms after crucial acoustic changes and because of its early onset in the present data set, we adjusted the main time window for analysis in a data-driven manner to the time period between 80–335 ms after acoustic divergence (Fig. 3c, the window is indexed by vertical lines). This windows includes both early maxima of RMS values collapsed across subjects and conditions. We also note that biphasic sMMN/m results, as revealed by the present data set, have been reported in several previous studies (e.g., Hanna et al. (2014), Hasting et al. (2007), Herrmann et al. (2009) and Pulvermüller and Shtyrov (2003)).

**Fig. 4:**
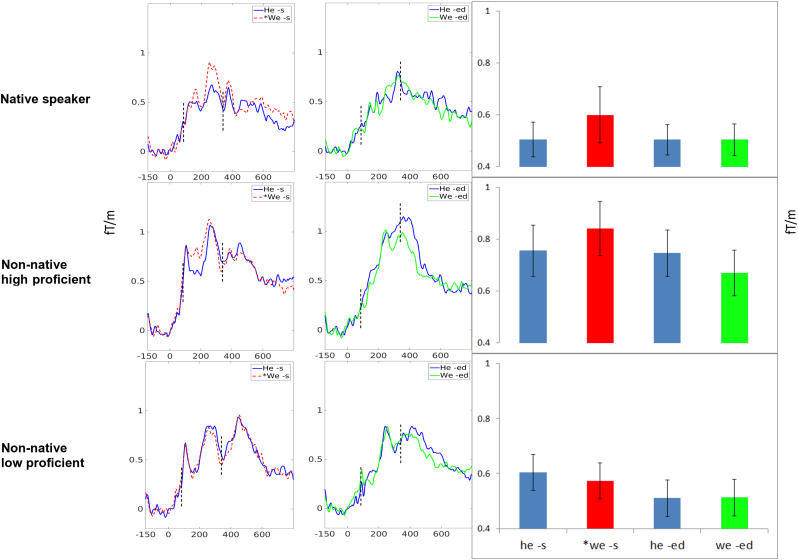
MMNm waveforms for the grammatical/ungrammatical he/*we –s conditions (first column in blue/*red) and the he/we –ed control conditions (second column in blue/green) as recorded in native speakers (top panels), high proficient non-natives (middle panels), and low proficiency non-natives (bottom panels). *y*-axes give magnetic field gradient in fT/m and *x*-axes give time in milliseconds. Bar charts in the right column show mean magnetic field gradient amplitudes taken within the 80–335 ms time window (again indicated by dashed lines on the waveforms) for each of the four conditions (with the ungrammatical one shown in red). Error bars represent standard error. (For interpretation of the references to color in this figure legend, the reader is referred to the web version of this article.)

Comparison of mean MMNm amplitudes in this time window across conditions showed that the MMNm for the ungrammatical affixed *we –s condition was larger than that for the corresponding grammatical he –s MMNm, but that differences between the control conditions, he –ed and we –ed, were comparatively small, and that this pattern was modulated across groups and hemispheres, such that the LP group tended to show weaker sMMNm, particularly in the right hemisphere. The NS and HP groups however both produced robust sMMNs (Fig. 4).

To investigate the statistical reliability of any between-group condition differences in MMN responses, signal space results were submitted to a repeated measures factorial ANOVA, with HEMPISHERE (left or right), ANTPOS (anterior or posterior), PROUNOUN (he or we), and INFLECTION (–s or –ed) as orthogonal two-level within-subject factors, and GROUP (NS, HP, LP) as a three-level between-subjects factor. There was an interaction of HEMISPHERE*PRONOUN*INFLECTION*GROUP (*F*(2, 39)=3.43, *p*=0.042), confirming the reliability of the observed differences. Planned comparisons were then carried out to test the hypothesis 1) that the HP group coheres with the NS group (NS+HP versus LP), thus indicating a proficiency difference, and the alternative hypothesis 2) that the two NNS groups would cohere instead (NS versus HP+LP), thus supporting a distinction between L1 and L2 learners, independent of proficiency. First, the HEMISPHERE*PRONOUN*INFLECTION*GROUP interaction with the group configuration of the first hypothesis was compared against the same interaction with the group configuration of the second hypothesis. This yielded a significant contrast (*F*(1,39)=3.43, *p*=0.042), indicating that one hypothesis explains the HEMISPHERE*PRONOUN*INFLECTION*GROUP variance significantly better than the other.

Further planned comparisons tested the first hypothesis grouping, NS+HP vs LP, and found a significant HEMISPHERE*PRONOUN*INFLECTION*GROUP interaction (*F*(1,39)=6.82, *p*=0.012). In the high performance (NS+HP) group alone, the interaction of PRONOUN*INFLECTION was not different across hemispheres (*p*=0.34). The PRONOUN*INFLECTION interaction collapsed across hemispheres was significant (*F*(1,39)=6.65, *p*=0.014). Finally, the ungrammatical *we –s condition in the NS+HP group tended to produce a stronger sMMNm than the grammatical he –s condition (*F*(1,39)=3.28, *p*=0.077) whereas the difference between control conditions (he vs. we –ed) was far from significant (*p*=.34). In the LP group alone, planned comparisons showed a significant HEMISPHERE*PRONOUN*INFLECTION interaction (*F*(1,39)=6.35, *p*=0.015) due to a right-hemispheric trend towards stronger MMNs in the grammatical “he –s” condition relative to the ungrammatical condition – the opposite pattern seen in grammatically proficient individuals. However, a post-hoc comparison of the PRONOUN*INFLECTION interaction in each hemisphere indicated it only trended toward significance in left (*F*(1,39)=1.17, *p*=0.28) and right (*F*(1,39)=2.69, p=0.1) hemispheres. The implications of these trends in the LP group are taken up in the discussion (4.2). As a test of the second hypothesis, that brain signatures of syntax processing differ independently of proficiency, the HEMISPHERE*PRONOUN*INFLECTION*GROUP interaction was tested with NS vs HP+LP, and did not yield significance (*F*(1,39)=2.26, *p*=.14). These results confirm a reflection of grammar proficiency in sMMN/m, but fail to support that this brain response indexes the NS/NNS distinction.

A correlational analysis where sMMN strength (*we –s MMN minus he –s MMN) in left and right hemispheres were compared to PC1 found that PC1 correlated with right hemisphere sMMN strength among NNS (*r*(26)=0.43, *p*=0.02). Right hemisphere sMMN strength correlated inversely with modified TROG errors (*r*(26)=−47, *p*=0.01). No such correlations obtained in the NS group, nor was there any AO correlation in the NNS group.

#### 3.3.3 Source space analysis

**Fig. 5:**
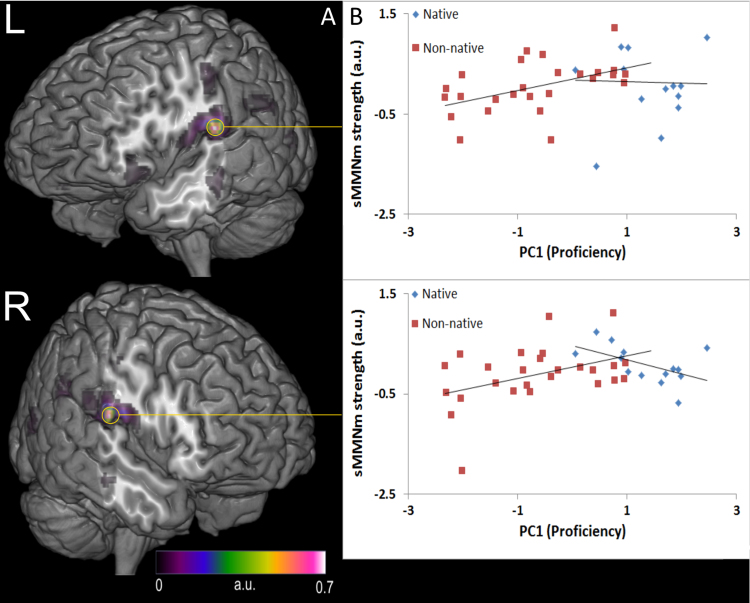
sMMNm sources and their relationship to psycholinguistic performance (values in arbitrary units, a.u.) A: Main sources (yellow circles) of the MMNm calculated across all conditions and across the 80–335 ms time window. The lateral sections of cortex have been removed to clearly show activation maxima. B: Strength of the left (top) and right (bottom) superior temporal main sources of the sMMNm (calculated as the difference MMNm for *we –s minus MMNm for he –s) plotted against psycholinguistic performance as captured by PC1. Nonnative speakers showed significant correlations between left- and right-hemispheric sMMN source strength and their individual performance on psycholinguistic tests. (For interpretation of the references to color in this figure legend, the reader is referred to the web version of this article.)

When collapsing the results of all subjects and conditions, the MMNm was localised to sources in bilateral middle temporal areas. Voxel values of the activity maxima, located in the superior temporal lobes in both hemispheres (MNI coordinates: −56–16 14 and 54–12 14, respectively) produced numerical results for the four conditions (he/we –s/-ed) similar to those obtained in signal space when comparing the three subject groups defined above (NS, HP, LP): both left and right superior temporal MMN sources tended to be stronger in the ungrammatical we –s conditions and weaker for he –s, he –ed, and we –ed conditions in both NS and HP groups, but not for LP. The interaction of PRONOUN*INFLECTION*GROUP was only marginally significant (*F*(1,37)=2.85, *p*=0.1). However, inspection of the distribution of sMMNm (*we –s minus he –s) across the non-native groups however showed that they follow a clear, linear path (Fig. 5b), and are therefore better analysed with a correlational model rather than a categorical factorial one. Data from the NNS group alone revealed that PC1 correlated with sMMNm strength in the left (*r*(24)=.48, *p*=0.012) and right (*r*(24)=.41, *p*=0.035) hemispheres. There was no relationship between PC1 and sMMNm in the NS group. Furthermore, the TROG error measure (see Methods) inversely correlated with sMMN source strength in the right hemisphere of NNS (*r*(24)=−.569, *p*=0.003), but not in the left one (*r*(26)=−16).

## 4 Discussion

This research has produced the following key findings: (1) the syntactic Mismatch Negativity, an early brain index of grammar processing not requiring focused attention, has been observed in response to subject–verb agreement violations perceived by some speakers of a late acquired second language (English). sMMNm responses similar to those in native speakers (NS) were present in non-native speakers with high grammar proficiency (HP), but not in low proficient non-native speakers (LP). (2) sMMNm strength in non-native speakers significantly correlated with the level of syntactic-grammatical knowledge and performance in their L2 as measured by online psycholinguistic syntax experiments (PC1). (3) The lack of verb inflection and subject–verb agreement in the NNS groups' L1 (Chinese) did not impede them from developing a fast, automatic capability for comprehending it. We discuss these points and others below in more detail.

### 4.1 sMMN in non-native speakers

As a wealth of psycholinguistic date demonstrate rapid, early syntactic processes within 200 ms upon presentation of critical stimulus onset, we explored the early brain index of grammar processing, the sMMN, and particularly its magnetic correlate, sMMNm. We found that the cortical sources of the sMMNm in bilateral superior-temporal cortex of non-native speakers of English correlated with their syntactic proficiency, as revealed by a newly explored assembly of online psycholinguistic tests. The earliness of the sMMN along with its sensitivity to grammar violations suggest that the earliest processes of syntactic parsing – or “first pass” syntax analysis (cf. Friederici, 2011) – are reflected by this measure. With regard to our non-native speakers, our data seem to show that these early processes of grammar analysis, more specifically the processes invoked by analysing subject–verb agreement, are developed to different degrees in more and less proficient L2 speakers and that the level of their processing of these constructions, as assessed by psycholinguistic tests, is reflected in the strength of brain activation in left and right posterior superior temporal gyri.

We are certainly not the first to explore linguistic ERP/F profiles across NS and late acquiring NNS groups. The majority of previous studies addressing this issue suggest that NNS may produce syntactic ERPs not qualitatively different from those in NS, and that proficiency in the L2 might be a critical factor determining the presence of these brain responses (Batterink and Neville, 2013a, Dowens et al., 2011, Dowens et al., 2009, Foucart and Frenck-Mestre, 2010, Frenck-Mestre et al., 2008, Hahne, 2001, Hahne and Friederici, 2001, McLaughlin et al., 2010, Pakulak and Neville, 2011, Sabourin and Stowe, 2008, Tanner et al., 2013a, Tokowicz and MacWhinney, 2005). All of these studies used late ERPs, for example the P600, which are well known to be under the strong influence of attention and task demand thus leaving it open to which degree grammar processes per se or processes following upon first-pass parsing were reflected. To our knowledge, early syntax indices such as the ELAN or sMMN/m were so far not shown to be present in speakers of a second, late acquired language to syntactic violations in that language. A handful of other studies have found that late acquiring NNS can also produce left anterior negativities (300–500 ms), e.g. in response to irregular German participles incorrectly given a regular inflection (Hahne et al., 2006), or in response to verb agreement errors, provided the participant is highly proficient (Ojima et al., 2005, Rossi et al., 2006), or to errors in an artificial language (Morgan-Short et al., 2012).

This present study now documents a brain correlate of L2 proficiency, which occurs earlier than these late components and possibly reflects the earliest stages of syntactic analysis. This ERP/F, the sMMN/m, is known to provide an early brain response to morphosyntactic agreement errors in native speakers, starting before 200 ms, and in some cases before 100 ms. In principle, the ELAN could also have provided information on early grammar processing, but in practice it has not so far been able to shed much light on L2 acquisition studies (Steinhauer, 2014, for review), perhaps owing to methodological shortcomings associated with ELAN experiments (Steinhauer and Drury, 2012). A further advantage of the sMMN may be that it emerges even when participants ignore the language stimuli and focus their attention elsewhere, thus avoiding the need for application of a language demanding task to low proficiency speakers. Finally, by using a restricted set of tightly matched stimuli, the MMN paradigm allows for exact tailoring of stimuli to the point in time where grammatical violations first become perceptible. This allows for precise timing of the brain response relative to the psycholinguistic processes involved. By contrast, experimental paradigms which do not control for this run the risk that the points in time when grammar violations are first recognisable are too variable across phrases, words and trials to yield consistent early differences in the ERP signal (for further discussion, see Pulvermüller and Shtyrov (2006)). In our present results, the first relevant grammar-related brain dynamics indeed began ca. 100 ms after the point in time when deviant stimuli first diverged from standard stimuli and thus when grammar violations could first be detected (i.e., when the –s morpheme started). Importantly, this early latency of grammar effects opens up a window on the very first neural stages of syntactic parsing, which were suggested by many previous behavioural studies (Marslen-Wilson, 1973, Marslen-Wilson, 1985, Marslen-Wilson and Tyler, 1975, Moss, 1997, Sereno et al., 2003, Trueswell and Kim, 1998, Tyler et al., 2002, Zwitserlood, 1989).

In summary, syntactic proficiency in L2 speakers was found to be reflected in the earliest brain correlate of one aspect of syntactic parsing, subject–verb agreement. Our behavioural and neurophysiological results indicate that NNS can acquire the capability to process local subject–verb agreement with a level of speed and automaticity that suggests nativelike processing capacity.

### 4.2 ‘Reverse’ sMMN

We observed that as proficiency (PC1) dropped, the sMMNm (MMN to *we –s minus MMN to he –s) also dropped. But this correlation did not cease once sMMNm levels reached zero. Rather, it reached well into negative values as proficiency decreased (Fig. 5), i.e. those non-native speakers with very low proficiency tended to produce stronger MMNm for grammatically *correct* conditions, in other words, a “reverse sMMN”. We did not predict this possibility, and the experiment was not designed to test it. We may however speculate that this reverse sMMN is an index of lexical access, suggesting less proficient learners of a language may sometimes process short phrases as single stored units rather than combinations of units. In other words, the phrase “he kicks” would be processed as a single lexeme or whole-form stored construction rather than a combination of two lexemes. It is becoming clear from MMN research that such whole form stored lexical items and constructions indeed elicit a different type of brain response pattern, namely larger MMNs to the stored form and smaller ones to unfamiliar combinations (e.g., pseudo-words and -constructions) (see Pulvermüller and Shtyrov (2006) for partial review). We have therefore two divergent patterns, where, when lexicality/whole-form storage applies, the MMN response to the correct condition is stronger than the MMN response to the incorrect condition, but the situation is reversed when a syntactic-grammatical combinatorial link exists between two stored items. These divergent patterns, which have already been used successfully to assess the neurophysiological correlates of whole-form storage vs. combination, most notably in linguistically ambiguous cases (Bakker et al., 2013, Cappelle et al., 2010, Hanna and Pulvermüller, 2014, Leminen et al., 2013, MacGregor and Shtyrov, 2013).

Our results hint at the intriguing possibility that this variation between arbitrary and rule-governed processes may exist not only across linguistic phenomena, but also across participants for the same linguistic phenomena, in present case for the parsing subject–verb agreement in short phrases. Applying this logic to non-native speakers with low grammar proficiency, their relatively enhanced brain response to grammatical combinations (he –s) suggests whole form storage of syntactic phrases. This suggestion fits well into neurological models of second language acquisition according to which grammar is first performed with the more flexible, faster learning declarative memory system, and only later, with growing proficiency, is the slower but more automatic procedural memory system brought into use (Paradis, 2009, Ullman, 2004). Future studies specifically designed to address this topic could shed more light on this issue.

### 4.3 Specific proficiency measures

A unique contribution of this study to the L2 neurophysiological literature is the exploration of online psycholinguistic measures for assessing L2 proficiency. These measures provided accuracy and response time data about the specific psycholinguistic processes and knowledge types also under investigation in the neurophysiological study, namely about the processing and representation of subject–verb agreement. Most previous L2 studies assessed proficiency either with subjective self-ratings or with standardised language tests. The former have the disadvantage that subjectivity and attitudinal factors can bias the results, whereas the latter are offline measures typically of a wide range of linguistic skills and may, in addition, be influenced not only by linguistic processes per se but, in addition, by more reflective post-comprehension processes. We used an offline grammar test (a modified version of the TROG) as an external validation of the psycholinguistic measures. While modified TROG scores correlated with right hemisphere sMMNm strength, they did not correlate at all in the left hemisphere, unlike the first principal component extracted from the psycholinguistic test data, PC1, which correlated with the strength of the main sMMNm sources in both hemispheres in the source space analysis. This is all the more remarkable when one considers how strongly PC1 and the modified TROG scores themselves correlated with each other (*r*=−.71 among NNS), indicating that the psycholinguistic measures are sensitive to a rapid, unreflective level of language performance that even specialised grammar tests are not sensitive to, and further that this performance may be seated primarily in left perisylvian language areas.

An area of potential further improvement is suggested in the lack of correlation between PC1 and sMMNm among the NS group. Another study has found performance differences within an NS group, correlating with educational attainment (Street and Dabrowska, 2010), so it would not be unreasonable to expect something similar here, as well. However, for a basic grammatical knowledge domain such as subject-verb agreement, nearly all NS would be near ceiling. Indeed, the range of our NS group on PC1 was smaller than that of NNS, and statistical analysis showed a significant differences in the variances (Levene *F*(1,40)=5.24, *p*=0.027). Future psycholinguistic grammar testing procedures may therefore aim at more appropriate mapping of the grammar knowledge spectrum available in L1 speakers.

Still our present pattern of results suggests that the combined psycholinguistic measures are the best predictor of the quality of brain mechanisms of specific types of grammar processing in speakers of an L2. The cross-validity shown by the significant correlations between on-line psycholinguistic tests of syntax and the early-latency automatic ERP measure sMMN suggests that the earliest grammar-related processes are monitored by these behavioural and neurophysiological measures. However, it is clear that our present results, now shown in a single study, call for replication and further validation in subsequent experiments. One question that arises addresses the relationship between early and late syntax responses, a question we cannot answer based on the present data, because no late syntax responses were elicited in our distraction oddball-like paradigm

### 4.4 Acquisition of L2 syntactic features not immanent to the L1

We found that Chinese speakers performed surprisingly well on tests of grammatical proficiency of a construction type not available in their L1, namely subject-verb agreement. Especially the neurophysiological responses, the MMNm to syntactic and asyntactic word strings, did not generally differ between L1 and L2 speakers. At first glance, these results appear different from the previous L2 ERP studies which investigated L1-L2 differences; these tended to find that acquisition of L2 syntactic features not in the L1 is more inhibited than features shared across L1 and L2. Closer examination of the studies however reveals no necessary contradiction. Tokowicz and MacWhinney (2005) only found inhibition of grammar learning where there is a conflict between the L1 and L2 grammatical features. In the case where the L2 feature simply did not exist in the L1 (in their case, gender agreement), NNS ERPs showed sensitivity to errors, which agrees with our present findings. Sabourin and Stowe (2008) and Foucart and Frenck-Mestre (2010) also found inhibited learning, but again this was not with an L2 feature absent in the L1, but rather with a shared feature that had conflicting ways of being realised between the L1 and L2. Therefore it appears that our results of similar neurophysiological indexes in L1 and L2 speakers of a feature absent in the L2 speakers' L1 are compatible with past research on L1 transfer/inhibition. However, it is surprising that the acquisition of a grammar feature not present in the native language seems to be almost native-like in late, successful L2 learners.

### 4.5 Handedness and age

All participants were strongly right-handed, but the NS group was more strongly lateralised than the NNS groups according to the Edinburgh inventory. Also, the general high proficient group (NS+HP) was somewhat younger and more right-handed than the LP group. Although these differences were statistically significant, we stress here their small scale; a 3 year difference on age, and a 15 point difference on laterality out of a 200 point scale. Therefore, while the groups were not perfectly matched on these factors, we think it unlikely they have substantially influenced the results in this case.

### 4.6 Between group signal strength differences

The HP group showed the highest overall signal strength, followed by the LP group, with the NS group producing the smallest MMNm. Generally, a strong co-determiner of signal strength in MEG is the interaction of head position, and cortical and cranial geometry of individual participants with the MEG sensors. The magnetic field weakens with the cube of distance, so very small differences in distance can make very big differences in signal strength. It is not clear whether the overall signal strength differences can be traced back to cognitive factors. However, as our main results build upon differential responses between conditions with similar stimuli and the presence, we believe that overall signal strength differences cannot account for the reported results.

### 4.7 Innovative nature of study

After a range of neurophysiological indexes of syntactic and semantic processing had previously been used to explore the brain basis of 2nd language processing, we performed the first study applying the sMMN paradigm in non-native speakers. As MMNm responses to linguistic stimuli, especially in non-natives with low L2 language proficiency, were quite variable, we chose a data-driven approach adjusting the main time window of analysis to the first double peak exhibited by the MMN response. Still, both the topography and early general time course of the MMN response were consistent with those of past linguistic MMN studies in native speakers. As a further innovation, a new index of syntactic performance was developed, using standard psycholinguistic tasks such as gating and speeded grammaticality judgement. The combination of on-line psycholinguistic measures using PCA represents a further innovative aspect, although the decision to divide subjects in proficiency groups by median-split was a post-hoc decision based on the shape of the data. However, as the main results from the present study emerged (not from factorial but) from correlation analyses – demonstrating a relationship between the left and right hemispheric sources of syntactically-elicited brain activity and syntactic proficiency as derived from psycholinguistic measures – we hope that our unexpected results can provide the seed for future research.

## 5 Conclusions

Grammatically proficient speakers of English as a late-acquired L2 showed native-like brain indices of grammar processing as reflected by the early and attention-independent syntactic MMNm, elicited by violations of subject–verb agreement. This suggests that L2 learners can acquire a sensitivity to aspects of grammar, here explored in the domain of local subject–verb agreement, that is difficult to distinguish at the neurophysiological level from that of native speakers. As a main key finding, we discovered that the performance on psycholinguistic grammar tests of individual subjects was reflected in the strengths of their brain-internal sources of neurophysiological activity elicited by grammatical violations. The better they performed, the stronger this “grammar brain activity” was. Comparisons between general syntactic proficiency assessment and tasks that are fine-tuned to local subject-verb agreement showed that only on-line psycholinguistic task performance predicted sMMNm production in both hemispheres. Our results suggest that the sMMNm can be produced even by those who began acquiring their L2 relatively late, and even in cases where the syntactic feature under investigation is not part of the L1. This may be difficult to explain for theories maintaining that late-acquired L2s must be fundamentally non-native-like in those aspects where their syntax diverges from L1.

## Acknowledgements and funding

We wish to thank Clare Cook, Oleg Korzyukov, and Maarten van Casteren for assistance in data collection, and two anonymous reviewers for many helpful suggestions on an earlier version of this work.

This research was supported by the Medical Research Council (MC_US_A060_0034, U1055.04.003.00001.01 to F.P., MC_US_A060_0043, MC-A060-5PQ90 to Y.S.), the Freie Universität Berlin, the Deutsche Forschungsgemeinschaft (Excellence Cluster Languages of Emotion, Project Pu 97/16-1 on “Construction and Combination”) to F.P. and J.H., and the Overseas Research Student Award Scheme, the Cambridge Trust, and the Language Learning Dissertation Grant to J.H.

## Figures and Tables

**Table 1:** Demographic Information.

	N	Age	Oldfield	Age of onset	N of males
**NS**	**14**	**23.9 (3.5), 19–32**	**93.9R (7.4), 77–100R**	**N/A**	**6**
**NNS**	**28**	**26.9 (3.4), 23–35**	**74.5R (25.4), 20–100R**	**11 (2.4), 6–16**	**10**
**HP**	**14**	**25.8 (2.9), 22–34**	**77R (24.4), 23–100R**	**10.8 (2.6), 6–16**	**4**
**LP**	**14**	**28 (3.6), 23–35**	**72R (27), 20–100R**	**11 (2.4), 7–14**	**6**
**NS+HP**	**28**	**25 (3.4), 19–34**	**86.7R (17.5) 23–100R**	**N/A**	**10**

Comparison of subject groups. Information about age, handedness (laterality indices according to the Edinburgh handedness inventory), age of second language learning onset and gender information (number of males) is presented for the groups of native speakers of English (NS) and Chinese non-native speakers of English (NNS). The latter group is broken down into low and high proficiency subgroups, NNS HP and NNS LP (see Section 3 for details on proficiency partition), as well as a general high proficiency group (NS**+**NNS HP). Means are given along with standard deviations in parentheses, followed by ranges.

**Table 2:** Stimuli.

	Standards (*p*=0.5)	Deviants –s (*p*=0.25)	Deviants –ed (*p*=0.25)
He	Standard 1: *He tick	**Deviant 1: He ticks**	**Deviant 4: He ticked**
(Block A)	Standard 2: *He pick	**Deviant 2: He picks**	**Deviant 5: He picked**
	Standard 3: *He kick	**Deviant 3: He kicks**	**Deviant 6: He kicked**
			
We	**Standard 1 We tick**	Deviant 1: *We ticks	**Deviant 4: We ticked**
(Block B)	**Standard 2: We pick**	Deviant 2: *We picks	**Deviant 5: We picked**
	**Standard 3: We kick**	Deviant 3: *We kicks	**Deviant 6: We kicked**

Standard and deviant stimuli used in the two blocks of the multi-feature MMN paradigm applied. In each block, frequent standard stimuli and rare deviants started with the same pronoun, followed by a verb stem, which, in the case of deviants, also carried an overtly realised affix (–s or –ed). Probabilities with which each stimulus type occurred are indicated in brackets.

## Footnotes

1 Gradiometers include: MEG0212, 0213, 0222, 0223, 0322, 0333, 0342, 0342, 0232, 0233, 0242, 0243, 1612, 1613, 1622, 1623, 1312, 1313, 1322, 1323, 1222, 1223, 1232, 1233, 1332, 1333, 1342, 1343, 2412, 2413, 2422, 2433.
